# Ammonium Transporter 1 (*AMT1*) Gene Family in Pomegranate: Genome-Wide Analysis and Expression Profiles in Response to Salt Stress

**DOI:** 10.3390/cimb47010059

**Published:** 2025-01-16

**Authors:** Fatima Omari Alzahrani

**Affiliations:** Department of Biology, Faculty of Sciences, Al-Baha University, Al-Baha 65729, Saudi Arabia; drfatimaomari@gmail.com or fsalomari@bu.edu.sa

**Keywords:** ammonium uptake, transport systems, AMT1s, phylogenetic analysis, pomegranate

## Abstract

Understanding the ammonium (NH_4_^+^) uptake and transport systems, particularly *AMT1* genes, is important for plant growth and defense. However, there is a lack of research on identifying and analyzing *AMT1* genes in pomegranate, emphasizing the need for further investigation in this area. Five *AMT1* genes (*PgAMT1-1* to *PgAMT1-5*) were identified, all of which contain the PF00909 domain, a feature of ammonium transporters. Various characteristics of these genes, including gene length, coding sequence length, and chromosomal locations, were examined. This study evaluated the isoelectric point, hydropathicity, conserved domains, motifs, and synteny of the PgAMT1 proteins. Phylogenetic analysis confirmed the homology of *PgAMT1* genes with previously reported *AMT* in Arabidopsis and tomato. The tissue-specific expression analysis of *PgAMT1* genes revealed distinct patterns: *PgAMT1-1* and *PgAMT1-2* were predominantly expressed in flowers, *PgAMT1-3* exhibited notable expression in roots, leaves, and flowers, *PgAMT1-4* was primarily expressed in leaf tissue, while the expression of *PgAMT1-5* was detected in both leaves and roots. The impact of salt-induced stress on *AMT1* gene expression was also examined, revealing that *PgAMT1-1*, *PgAMT1-2*, and *PgAMT1-4* expression is reduced under increased salt stress. These expression modifications can help regulate NH_4_+ assimilation in conditions of elevated salinity, maintaining cellular homeostasis and ion balance. This study contributes to the comprehensive identification of the *AMT1*s gene family in pomegranate; however, further research on the functional characterization of the identified *PgAMT1*s is needed.

## 1. Introduction

Nitrogen is an essential element for the growth and development of plants and a significant factor to consider in assessing productivity. Higher plant roots typically acquire inorganic nitrogen from two primary sources, namely, ammonium (NH_4_+) and nitrate (NO_3−_), which are commonly found in natural and agricultural settings [1,2,3]. When plants are experiencing nitrogen deficiency, they exhibit a preference for NH_4_+ uptake over nitrate (NO_3−_) due to the former’s lower energy requirement for assimilation [4,5,6,7]. Whilst NH_4_+ uptake is known to be more efficient, usually ranging from 20 to 200 μM in agricultural soils, it should be noted that excessive uptake of NH_4_+ by the plant can result in toxicity in the absence of nitrate [8,9].

The uptake of NH_4_+ is suggested to occur via ammonium transporters (AMTs) that are categorized under the ammonium transporter/methylammonium permease/Rhesus (AMT/MEP/Rh) family [5,10,11,12]. This particular family of transporters exhibits a number of shared characteristics, including a marked affinity for ammonium (with a Km (Michaelis constant) in the micromolar range; this suggests that they can effectively transport ammonium ions, even when they are present in low concentrations and a pronounced selectivity for this particular ion. Specifically, these transporters can only transport ammonium and its methylated analog, methylammonium (MeA), while being incapable of transporting other monovalent cations. In addition, these transporters reach saturation at ammonium concentrations that do not exceed 1 mM [6,10,13].

Despite the observed similarities, there remains ambiguity regarding the specific chemical species that is being transported, namely, NH_4_+ or ammonia (NH_3_), as well as the transport mechanism that these proteins represent, whether it be a channel or a transporter [14,15,16]. The investigation of the crystal structure of AmtB from *Escherichia coli* and Amt1 from *Archaeoglobus fulgidus* has led to significant advancements in comprehending ammonium transporters [14,17,18,19]. The aforementioned research has indicated that the ammonium transporters exhibit homotrimeric conformation, wherein each monomer comprises a central hydrophobic channel that is postulated to serve as the conduit for the transportation of uncharged ammonia (NH_3_). The uptake of NH_4_+ in plants is facilitated by the ammonium transporter (AMT) family. Ammonium absorption in plants is mediated by the ammonium transporter (AMT) family. In diverse plant species, including Arabidopsis, rice, tomato, and maize, *AMT* gene members have been subjected to cloning and functional characterization. On the basis of their respective amino acid sequences, the six AMT proteins encoded by the Arabidopsis genome can be classified into two subfamilies, AMT1 and AMT2 [6,20,21]. The initial stage of nitrogen assimilation involves the conversion of NO_3−_ to NH_4_+, succeeded by the integration of NH_4_+ into amino acids [22,23]. Root cells utilize specific transporters in the plasma membrane to absorb these ions [13]. The genes present in the *AMT1* family are responsible for encoding proteins that exhibit a notable capacity for transporting NH_4_+ ions with a high degree of affinity. The uptake capacity for NH_4_+ in nitrogen-deficient roots is attributed to AtAMT1-1 and AtAMT1-3, which account for 30–35% of the capacity, and AtAMT1-2, which accounts for 18–26% [24,25,26]. In addition, the expression *AtAMT1-4* specifically in pollen plays a role in the nitrogen nutrition of pollen through the uptake or retrieval of NH_4_+ [26,27].

Pomegranate (*Punica granatum* L.) is cultivated in tropical and subtropical climates [28,29]. The plant has garnered significant interest owing to its fruit’s potent antioxidant constituents, health-promoting properties, medicinal attributes, and anthocyanin content [30]. The plant is known for its ability to tolerate high levels of salt [28], and it was suggested that pomegranate exhibits potential as a viable model plant for deciduous fruit trees, enabling the exploration of their responses to diverse environmental stressors [31].

The attributes of the *AMT1* genes have yet to be documented in pomegranate. Thus, the present investigation conducted a thorough examination of the *AMT1* gene family in pomegranate at a genome-wide level. This study identified the *AMT1* gene family in pomegranate, conducted sequence and phylogenetic analyses, examined gene duplication and chromosomal locations, and analyzed the characteristics of the *PgAMT1* genes and proteins. This study also aimed to examine the expression profiles of the *PgAMT1* gene family across various tissues and analyze the gene expression of the *PgAMT1* family in leaves under salt stress. The results obtained from this study have the potential to serve as a reference for future research on the detailed functions of the *AMT1* genes.

## 2. Materials and Methods

### 2.1. Plant Materials

The pomegranate seeds (local cultivar) were collected from the Seed and Seedling Center, Al-Baha, Saudi Arabia. The seeds were planted in commercial potting soil (Almarai, Saudi Arabia) in a growth chamber under 26 °C during the day and 22 °C at night. The humidity level was maintained at 60%, and the seeds were exposed to 14 h of light followed by 10 h of darkness each day. A total of 24 pots, each approximately 5 inches diameter in size, were arranged in a completely randomized manner across three blocks. Each block contained eight pots, and every two pots within a block were treated as biological replicates.

After three months of growth, seedlings (about 12 inches in height) were subjected to different concentrations of NaCl (0 mM as a control, 100 mM, or 200 mM) every six days. A saucer was positioned beneath the containers to maintain the soil’s moisture. After around 20 days of treatment, the detrimental effects of salt on the pomegranate plant were evident. As a result, all leaves were collected from each plant individually during harvesting.

### 2.2. Identification of the AMT1 Family Genes in P. granatum and Sequence Analysis

*P. granatum*’s genomic sequence and annotation were retrieved from the NCBI genome database (https://www.ncbi.nlm.nih.gov/assembly/GCF_007655135.1), accessed on 1 December 2024. To identify the *AMT1* gene family in *P. granatum*, the information regarding the *AMT1* gene family of *Arabidopsis thaliana* was obtained from The Arabidopsis Information Resource (TAIR) database (https://www.arabidopsis.org/ accessed on 1 December 2024), and used as a query in the HMMER (3.3.2) software for building the hidden Markov model profile. This model was used to search for the AMT1 proteins of *P. granatum* with the HMMER software. The SMART and Pfam databases were utilized to confirm AMT1 members among the candidates. This was performed to eliminate redundant sequences and ensure that only those sequences containing the (PF00909) domain were retained (https://www.ebi.ac.uk/interpro/search/text/, accessed on 1 December 2024) (UniProt, 2021). *P. granatum’s* sequence and annotation were retrieved from the NCBI genome database (https://www.ncbi.nlm.nih.gov/assembly/GCF_007655135.1, accessed on 1 December 2024).

### 2.3. Phylogenetic Analysis, Gene Duplication and Chromosomal Location

The construction of the phylogenetic tree involved the utilization of protein sequences derived from *AMT1* genes originating from *P. granatum*, Arabidopsis (*A. thaliana)*, and tomato (*Solanum lycopersicum)*. The alignment of all AMT1 sequences was performed using MAFFT v7.402 [32]. The phylogenetic analyses were conducted utilizing the raxmlGUI software with 1000 bootstrap repetitions and the GTR+G model for maximum likelihood estimation [33]. To obtain the Ka, Ks, and Ka/Ks ratios, homologous genes from multiple species undergo sequence alignment. The calculations were conducted utilizing the TBtools software. The divergence time (T) was determined using the formula T = Ks/(2 × 6.1 × 10^−9^) 10^−6^ Mya. Paralogous genes with a Ka/Ks ratio of more than 1 were very positive, about 1 was neutral, and less than 1 was purifying. Based on data from the *PgAMT1* family of genes, chromosomal distribution and collinear *PgAMT1* gene maps were created using the TBtools tool version II [34]. The synteny analysis between the *AMT1* gene in pomegranate, Arabidopsis, and tomato was conducted using the TBtools program. Subsequently, the results were visualized through a dual Synteny Plot.

### 2.4. Sequence Analysis and Characteristics of Genes and Proteins

Using the Conserved Domain Database (CDD) (https://www.ncbi.nlm.nih.gov/cdd, accessed on 1 December 2024), all detected PgAMT1 protein sequences were confirmed by a conserved domain search (accession number: PF00909) [35]. Gene Structure Display Server 2.0 (GSDS; http://gsds.cbi.pku.edu.cn/, accessed on 3 December 2024) was used to present *PgAMT1* gene structures. Using the Plant-mSubP online tool https://bioinfo.usu.edu/Plant-mSubP/, accessed on 3 December 2024, the predicted subcellular localizations of AMT1 proteins were discovered [36]. TMHMM Server v.2.0 was used to predict transmembrane helices in PgAMT1 proteins (http://www.cbs.dtu.dk/services/TMHMM-2.0/, accessed on 3 December 2024) [37]. The analysis of conserved motifs was conducted using MEME server v 5.0.5 (http://meme-suite.org/tools/meme, accessed on 3 December 2024) with the default parameters [38]. Information concerning the isoelectric point (IP) and relative molecular mass of the PgAMT1 proteins was obtained from the website https://web.expasy.org/compute_pi, accessed on 5 December 2024.

### 2.5. Expression Profile Analysis of the PgAMT1s Gene Family in Different Tissues

For the patterns of *PgAMT1*s expression in different tissues and organs, the NCBI database’s Illumina sequencing platform (http://www.ncbi.nlm.nih.gov/, accessed on 1 December 2024) was used. Three different pomegranate tissue samples were successfully used to obtain transcript data (SRR5279396, SRR5279395, and SRR5279397). The RNA-Seq was screened for quality using FASTQC [39]. Trimming adaptors from the dataset were carried out using the Trimmomatic tool [40]. Using HISAT2 (Version 2.2.1), the RNA-seq was then mapped to the pomegranate reference genome (ASM765513v2) [40,41,42]. Using kallisto (Version 0.48.0), transcript abundances from RNA-Seq data were quantified [41]. Heatmaps were created using TBtools to display the values of log2 (TPM+1).

### 2.6. RNA Isolation and Gene Expression Analysis of the PgAMT1s Gene Family in Leaves Under Salt Stress

RNA was extracted from leaves using Trizol reagent in accordance with the guidelines provided by the manufacturer. The integrity of RNA was assessed using gel electrophoresis. The quantification of RNA levels in the samples was performed using the NanoDrop 2000 Spectrophotometer (Thermo Scientific, Loughborough, UK). RT-qPCR was performed using the Roche Light Cycler 96 System. Using the Luna Universal One-Step RT-qPCR Kit from NEB, the expression of the *PgAMT1-1*, *PgAMT1-2*, *PgAMT1-4,* and *PgAMT1-5* genes was measured in 10 ng RNA samples. For RT-qPCR investigation, the genes’ forward and reverse primers (Table S1) were designed. The 2^−∆∆CT^ method was employed to calculate the relative expression of *PgAMT1*s in comparison to the expression levels of the housekeeping gene ACTIN (NCBI: OWM65733). The cycle threshold (Ct) values were derived from three independent biological replicates. To determine the statistical significance of the variation in gene expression between control and stress conditions, a two-tailed unpaired *t*-test with a * *p* < 0.01 was performed with Excel (version 4210).

### 2.7. Statistical Analysis

Statistical analyses were conducted to assess the significance of changes in gene expression levels under different salt stress settings. The data were analyzed utilizing a two-tailed unpaired *t*-test to compare the expression levels of *PgAMT1* genes between the control group (0 mM NaCl) and the treated groups (100 mM and 200 mM NaCl). A portability value below 0.01 (* *p* < 0.01) was deemed statistically significant. The results were displayed as mean values accompanied by standard error bars to demonstrate the variability among the biological replicates. The figure was drawn using Excel (version 4210).

## 3. Results

### 3.1. Identification of the AMT Family Genes in P. granatum and Sequence Analysis

The pomegranate genome was analyzed using the HMM profile “PF00909” to find the *AMT1* genes. The results revealed five probable PgAMT1 proteins and their associated encoding genes (Table 1). The length of the encoded proteins varied between 460 and 514 amino acids. The isoelectric points (Ip) of the PgAMT1s exhibited a range of values, spanning from 5.56 to 7.17. The molecular weight exhibited a range of values, spanning from 49.65 kDa to 54.88 kDa. The analysis of transmembrane domains in the PgAMT1 proteins revealed that all PgAMT1 proteins possess nine transmembrane helices, except for PgAMT1-1, which contains 11 domains. Based on subcellular localization predictions, it was determined that the PgAMT1 proteins, namely PgAMT1-1, PgAMT1-3, and PgAMT1-4, are localized on the plasma membrane, whereas PgAMT1-2 and PgAMT1-5 are localized within the vacuole.

### 3.2. Phylogenetic Analysis, Gene Duplication and Chromosomal Location

**Figure 1:**
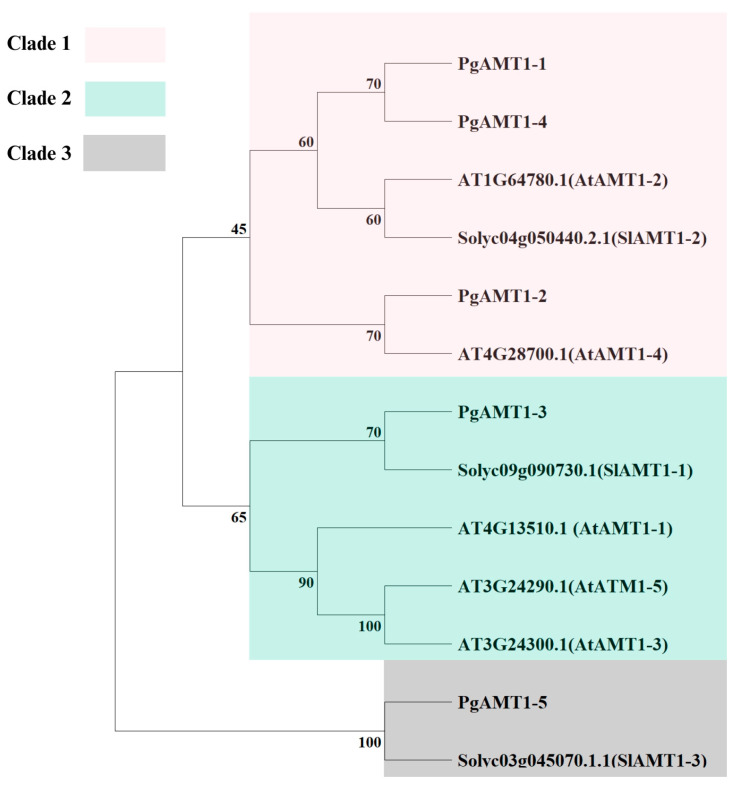
The phylogenetic tree of the AMT1s proteins from *Punica granatum*, *Arabidopsis thaliana,* and *Solanum lycopersicum*. The clades are represented using a range of distinct colors. The values of bootstrap are provided.

**Figure 2:**
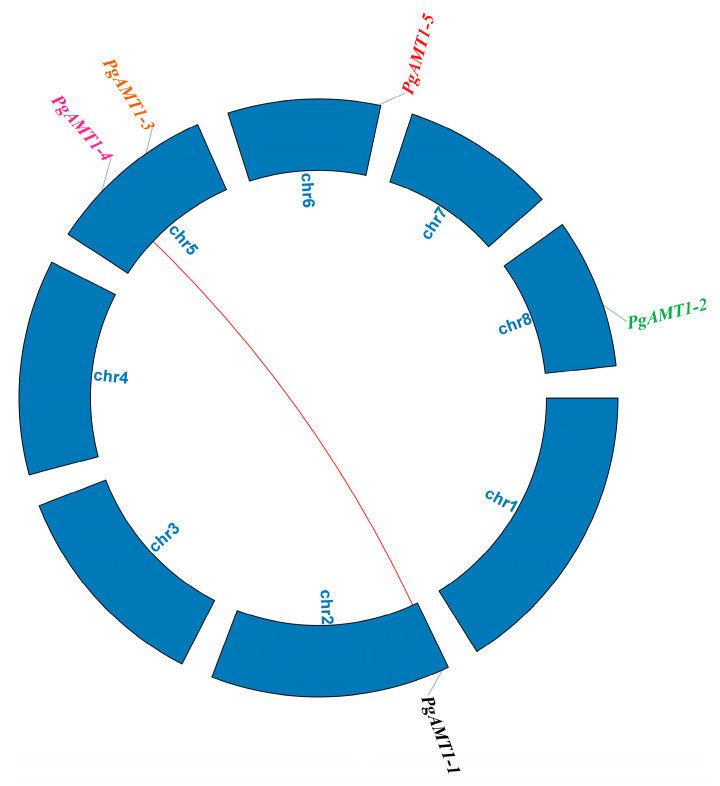
Chromosomal location and gene duplication of *PgAMT1* gene family. The red line indicates the synteny between *PgAMT1-1* and *PgAMT1-4* genes.

**Figure 3:**
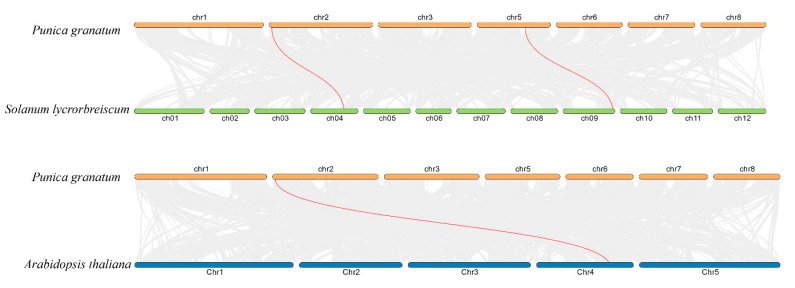
The synteny analysis of the *AMT1* gene family members in three plant species, *Punica granatum*, *Arabidopsis thaliana*, and *Solanum lycopersicum*. The background of the image displays grey lines that show the collinearity of the complete genome, whereas the red lines specifically demonstrate the collinearity of the *AMT1* genes.

Phylogenetic analysis of AMT1s in pomegranate, Arabidopsis, and tomato showed that the AMTs are divided into three clades (Figure 1). Each clade contains AMT1 proteins from different plant species. Clade 1 showed the phylogeny of PgAMT1-1, PgAMT1-2, and PgAMT1-4 with SlAMT1-2, AtAMT1-2, and AtAMT1-4. Clade 2 showed the phylogeny of PgAMT1-3 with SlAMT1-1, AtAMT1-1, AtAMT1-3, and AtAMT1-5. Clade 3 PgAMT1-5 with SlAMT1-3. The pomegranate genome has five *PgAMT1* genes, which are distributed across four chromosomes. Chromosome 5 has two *PgAMT1* genes, *PgAMT1-3* and *PgAMT1-4*. Chromosome 2 harbors *PgAMT1*-1 genes, while Chromosomes 6 and 8 contain the *PgAMT1-5* and *PgAMT1-2* genes, respectively (Figure 2). In order to investigate the correlation between *PgAMT1* genes and duplications within the pomegranate genome, a synteny analysis was conducted on the *AMT1* gene family in pomegranate. The genes of *PgAMT1*s were annotated on the physical map of the pomegranate chromosomes using location information obtained from NCBI. Based on the synteny analysis, it is predicted that *PgAMT1-1* and *PgAMT1-4* exhibit segmental duplication (Figure 2). The Ka/Ks ratio of the *PgAMT1-1* and *PgAMT1-4* pair was less than one, indicating a purifying selection. The values of ka, ks, and ka/ks are presented in Table 2. A comparative analysis was carried out to examine the correlation between *AMT1* genes in pomegranate, Arabidopsis, and tomato using a synteny analysis. The analysis conducted in this study identified the existence of a single pair of syntenic *AMT1* genes between pomegranate and Arabidopsis, as well as two pairs of syntenic *AMT1* genes between pomegranate and tomato (Figure 3).

### 3.3. AMT1 Genes and Proteins Structures

**Figure 4:**
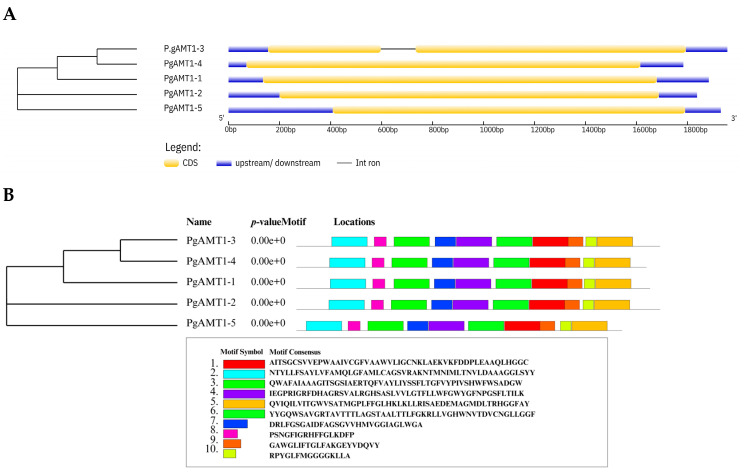
(**A**). The *PgAMT1* genes’ intron/exon structures are shown. Exons are represented by the yellow boxes, introns by the black lines, and untranslated regions (UTRs) by the blue boxes. (**B**). PgAMT1 proteins’ conserved motifs. Different preserved motifs were represented by various colored boxes.

All *PgAMT1* genes possess a single exon, with the exception of *PgAMT1-1*, which contains two exons and one intron (Figure 4A). The motif distribution analysis revealed the presence of ten conservative motifs in PgAMT1 proteins. All PgAMT1 proteins exhibit identical motifs, as illustrated in Figure 4B.

### 3.4. Expression Profile Analysis of the PgAMT1s Gene Family in Different Tissues

**Figure 5:**
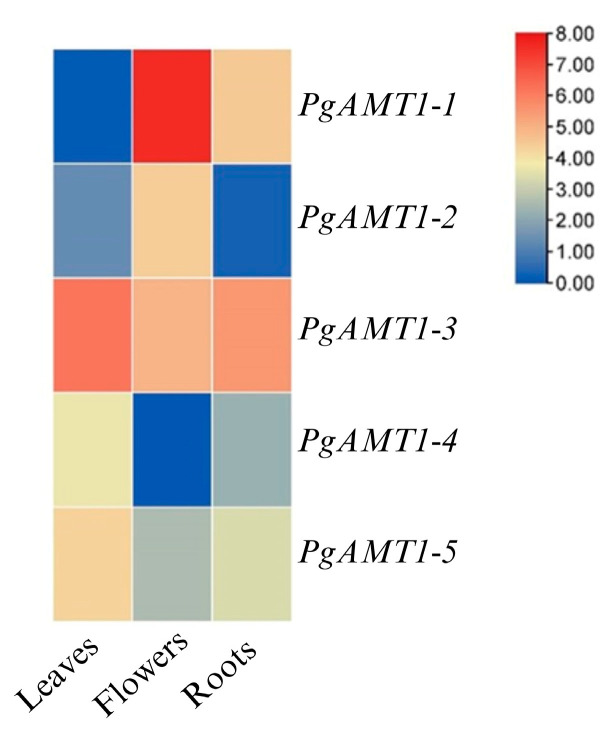
Expression profiles of *AMT1* genes in *Punica granatum* in root, flower, and leaves. The transcriptomic data were normalized via log2 (TPM + 1) to generate a heatmap.

*PgAMT1-1* exhibited high expression levels in flowers, moderate levels in roots, and no expression in leaves as illustrated in Figure 5. In contrast, *PgAMT1-2* was found to express solely in flowers among the analyzed tissues. *The pgAMT1-3* gene demonstrated high expression across the roots, leaves, and flowers of the pomegranate plant. *PgAMT1-4* showed expression in leaves with limited expression in roots.

### 3.5. Expression Analysis of the PgAMT1s Gene Family in the Leaves Exposed to Salt Stress

**Figure 6:**
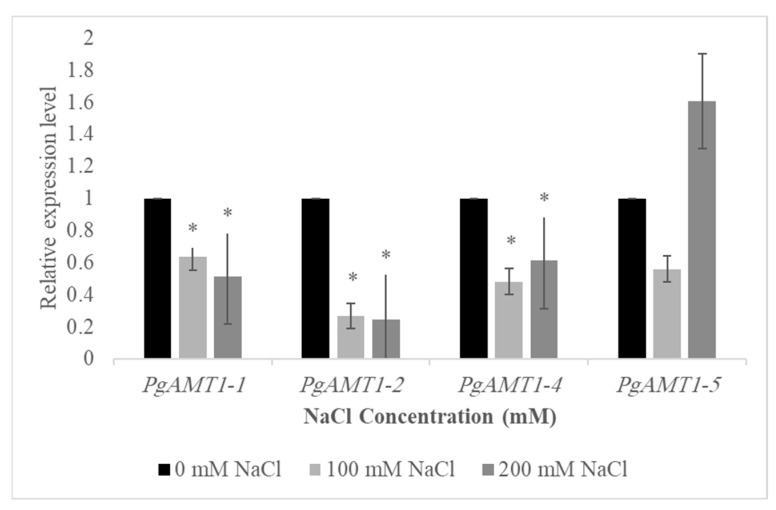
Expressions of *PgAMT1-1*, *PgAMT1-2*, *PgAMT1-4*, and *PgAMT1-5* in *Punica granatum* under salt stress (100 mM NaCl, 200 mM NaCl compared to the control 0 mM NaCl) by RT-qPCR. The two-tailed unpaired *t*-test is used to obtain the *p*-value (* *p* < 0.01) and significant differences are denoted by an asterisk(s). The error bar indicates the standard error.

The expression of the *PgAMT1-1* gene showed a decrease compared to the control, indicating a reduced response to this salt concentration. In a similar manner, the levels of *PgAMT1-2* and *PgAMT1-4* decreased when exposed to a concentration of 100 mM NaCl. Interestingly, the expression of *PgAMT1-5* exhibited a modest increase under the same conditions (100 mM NaCl). The elevated concentration of NaCl (200 mM) resulted in a subsequent reduction in the expression of *PgAMT1-1*, *PgAMT1-2*, and *PgAMT1-4*. Nevertheless, the level of *PgAMT1-5* expression showed an elevation in response to a concentration of 200 mM NaCl (Figure 6).

## 4. Discussion

During the development period, plants have a higher demand for nitrogen (N) compared to other essential nutrients. Nevertheless, the overall nitrogen use efficiency among plants is considerably low [43,44,45,46]. In addition, it has been observed that around 70% of nitrogen fertilizers applied are not absorbed by plants and consequently become waste [43,44,45,46]. This inefficiency highlights the need for better nitrogen management measures in agricultural activities. Ammonium (NH_4_+) is often preferred by plants among the many forms of nitrogen due to its reduced energy required for assimilation relative to nitrate (NO_3−_). The absorption of NH_4_+ across plant membranes is mediated by ammonium transporters (AMTs) [47]. A total of five *AMT1* genes were characterized from the pomegranate genome that are also found in other species such as Arabidopsis, rice, and tomato [13,20,48,49,50].

The structural organization of *AMT1* genes is influenced by evolutionary divergence, gene duplication events, and alternative splicing, which contribute to the functional variety within this gene family. There is considerable variety in the presence of introns and exons among species, with the majority of *AMT1* genes lacking introns, except for particular cases, such as *PgAMT1-1*, which contains a single intron. This aligns with previous research indicating that the majority of *AMT1* genes lack introns, with the exception of a few instances, such as *LjAMT1-1* in *Lotus japonicus* and *SlAMT1-2* in tomato [49,51]. This structural diversification may represent the adaptation mechanisms utilized by various plant species to maximize nitrogen uptake under diverse environmental situations. Introns exert a huge energy burden on cells. Hence, intronless genes are more efficient [52].

The evolution of gene families is affected by gene duplications, including whole-genome, segmental, and tandem duplications, which facilitate functional diversification and adaptation to environmental changes [53,54,55]. The lack of tandem duplications in the *PgAMT1* genes of pomegranate indicates that the expansion of this gene family has predominantly resulted from segmental duplication [56]. The Ka/Ks ratio for *PgAMT1-1* and *PgAMT1-4* has been examined, demonstrating that a purifying selection has occurred, suggesting that these genes have been conserved under evolutionary pressures, maintaining crucial activities in ammonium transport [57].

Phylogenetic analysis further explains the evolutionary relation of AMT1 proteins across many plant species. Studies have demonstrated the classification of the AMT1 protein family into multiple clades, each reflecting a lineage adapted to particular environmental conditions and nutrient demands [58,59]. Similarly, the finding of three clades within the AMT1 proteins of pomegranate, Arabidopsis, and tomato may indicate that diversification within this protein family is governed by the distinct nitrogen requirements of various plant species. Therefore, understanding the evolutionary dynamics and functional roles of *AMT1* genes can help design strategies to maximize nitrogen uptake and utilization, which will ultimately support sustainable agriculture practices.

This work also highlights the significant functional diversity of *PgAMT1* genes through tissue-specific expression patterns. *PgAMT1-1* demonstrated a role in both the reproductive and vegetative phases. The essential function of *PgAMT1-1* in the reproductive phase may be highlighted by the increased expression in flowers, with amino acid synthesis and secondary metabolite biosynthesis likely being facilitated. The expression of *PgAMT1-1* found in roots suggests a role in nutrition absorption. The potential function of *PgAMT1-2* in the reproductive stage is highlighted by its notable expression in flowers. Hence, *PgAMT1-2* may support flower development, which is consistent with studies in other species where *AMT1* genes have been associated with reproduction processes [60]. The expression profile of *PgAMT1-3* in roots, leaves, and flowers indicates a crucial involvement in plant physiology. The expression of *AMT* genes in cotton is controlled by nitrogen availability and differs among various plant tissues. *GhAMT1.3* in cotton was shown to be significantly expressed in roots and leaves during nitrogen deprivation and was subsequently downregulated upon the resumption of ammonium supply. This suggests that *AMT* genes are essential in the plant’s response to nitrogen supply, enabling ammonium absorption and distribution to different tissues, such as roots and leaves [61]. The expression of *PgAMT1-5* in both leaves and roots emphasizes its possible function in regulating nitrogen uptake and assimilation across diverse plant tissues.

The study of AMT1s in plants has received a lot of attention because these proteins play such an important role in nitrogen uptake under salt stress [62]. Salt stress can hinder the uptake, metabolism, and assimilation of nitrogen. The competition between chloride ions (CL-) and nitrate ions (NO_3_-) might restrict the uptake and utilization of nitrogen in plants that are experiencing salt stress [63]. The results of the current study demonstrate that *PgAMT1-5* shows overexpression in response to salt stress, whilst other *PgAMT1* genes show downregulation, indicating a complex adaptive response that might improve the plant’s resistance to elevated salinity. The fluctuating expression of *PgAMT1* genes in response to different NaCl concentrations highlights the intricate regulatory strategies that plants employ to maintain nitrogen balance when faced with abiotic stress. The observed decrease in *PgAMT1-1*, *PgAMT1-2*, and *PgAMT1-4* levels in response to increased salinity aligns with previous studies that have documented similar trends in various species. For example, the expression of *SlAMT1-2* in tomatoes showed a significant reduction under salt stress, indicating a possible similarity in the response of *AMT1* genes among various plant species [49]. This downregulation may serve as a protective mechanism to limit ammonium absorption, thereby preventing potential toxicity and maintaining cellular ion balance in saline environments. The increased expression of the *PgAMT1-5* gene in response to differing concentrations of NaCl (100 mM and 200 mM), despite the lack of statistically significant differences in expression levels compared to the control condition, may indicate a role in the improvement of salt tolerance in the pomegranate plant. This observation aligns with similar research in other species, such as *Populus simonii*, where the *PsAMT1.2* gene exhibited elevated expression under salt stress, enhancing ammonium absorption and enhancing salt stress tolerance [64]. However, further research may be required to explore the molecular processes governing the response of *PgAMT1-5* to salt stress.

The differential regulation of *PgAMT1* genes highlights the complexity of nitrogen metabolism in plants facing abiotic stresses. This suggests that targeted research on these transporters could yield valuable insights into enhancing crop resilience in saline environments. Future investigations may focus on elucidating the molecular mechanisms that regulate *PgAMT1* expression, as well as exploring the potential applications of these findings in agricultural practices aimed at improving nitrogen use efficiency and enhancing salt tolerance in crops. The integration of genomic, transcriptomic, and physiological approaches will be essential for deepening our understanding of how plants respond to environmental stresses and for optimizing agricultural productivity in the face of climate change.

## 5. Conclusions

Throughout the developmental phase, plants exhibit an increased need for nitrogen (N) when compared to other important nutrients. NH_4_+ and NO_3_- serve as essential nitrogen sources that facilitate optimal growth and development in plants. The process of NH_4_+ absorption in plants is facilitated by AMT1 proteins. The pomegranate genomes were found to possess a total of five *AMT1* genes. The structure and organization of *AMT1* genes can vary among plant species, and gene duplications have contributed to the expansion of the *AMT1* gene family. Phylogenetic analyses have revealed the diversification of AMT1 proteins in response to different nitrogen availability and utilization strategies. *PgAMT1* genes exhibit tissue-specific expression patterns, indicating their significance in nitrogen absorption and transport. Salt stress can affect the expression of *PgAMT1* genes, with some genes being upregulated and others being downregulated. These changes in expression may help plants adapt to salt-induced stress and maintain nitrogen metabolism. However, the regulation of *PgAMT1* expression in response to salt stress is complex and influenced by various factors. Further research is needed to explore the mechanisms underlying the expression and regulation of *AMT1* genes and their potential applications in agriculture. In addition, future studies should incorporate the quantification of ammonium concentrations alongside gene expression analysis.

## Figures and Tables

**Table 1:** The identification of the members of the *AMT1* gene family in pomegranate and their sequence analysis.

Locus Id	Accession Id	Gene Name	Gene Length	CDS (bp)	Protein Length (A.A)	Protein Molecular Weight (KDa)	IP	No of Exons	Cellular Localization	Number of Transmembrane Helices
LOC116195843	XP_031381085.1	*PgAMT1-1*	1884 nt	1545	514	54,881.87	5.84	1	Cell membrane	11
LOC116187132	XP_031371593.1	*PgAMT1-2*	1838 nt	1488	495	52,677.53	6.36	1	Vacuoles	9
LOC116208366	XP_031397616.1	*PgAMT1-3*	1821 nt	1503	500	53,436.31	6.36	2	Cell membrane	9
LOC116209315	XP_031398779.1	*PgAMT1-4*	1784 nt	1545	514	54,883.71	7.17	1	Cell membrane	9
LOC116210263	XP_031399970.1	*PgAMT1-5*	1931 nt	1382	460	49,655.12	5.56	1	Vacuoles	9

**Table 2:** Ka/Ks ratios and estimated divergence time for paralogous *PgAMT1-1* and *PgAMT1-4* genes.

Seq_1	Seq_2	Ka	Ks	Ka/Ks	Mya
*PgAMT1-1*	*PgAMT1-4*	0.159511	1.280654	0.124555	104.9716

Mya: million years ago.

## Data Availability

Data are available on request.

## Supplementary Materials

The following supporting information can be downloaded at https://www.mdpi.com/article/10.3390/cimb47010059/s1.
