# Supplementary material for: Ammonium Transporter 1 (AMT1) Gene Family in Pomegranate: Genome-Wide Analysis and Expression Profiles in Response to Salt Stress
Source: Curr Issues Mol Biol. 2025 Jan 16;47(1):59. doi: 10.3390/cimb47010059 (PMC11764171; doi:10.3390/cimb47010059)
Supplement: Supplementary file 1 [file cimb-47-00059-s001.zip › cimb-3419440-supplementary.pdf]

Table S1: Primers used for RT-qPCR analysis of genes.

Primer' name	Sequence
PgAMT1-1F	GGCAGCCCAAATCATACAAATC
PgAMT1-1Rev	CGAGATCCTCAACAGCTTCATC
PgAMT1-2F	GCAGCTTCTTCTACTACCTCTTC
PgAMT1-2Rev	ATGACTGGGACGGAAACTTG
PgAMT1-4F	GGCCTCTCCTATTACCTCTTTG
PgAMT1-4Rev	GTAGACGCAGGGTAGTCTTTC
PgAMT1-5F	ACACCATCTACCTCCTCTTCTC
PgAMT1-5Rev	GAGCATGATGTTCATGGCATTC
Pgactinfor	AGTCCTCTTCCAGCCATCTC
Pgactinrev	ACTGAGCACAATGTTTCCA
